# Supplementary material for: The Immune Signature of CSF in Multiple Sclerosis with and without Oligoclonal Bands: A Machine Learning Approach to Proximity Extension Assay Analysis
Source: Int J Mol Sci. 2023 Dec 21;25(1):139. doi: 10.3390/ijms25010139 (PMC10778830; doi:10.3390/ijms25010139)
Supplement: Supplementary file 1 [file ijms-25-00139-s001.zip › ijms-2727148-supplementary.pdf]

Supplementary material

Table S1. List of the biomarkers measured by means of PEA technique (inflammation panel) in the CSF of recruited patients. Biomarkers are listed according to the alphabetical order of the abbreviations. Proteins with a call rate <95% (excluded from further analysis) are indicated.

Abbreviations	Full or alternative names	Call rate < 95%
**ADA**	Adenosine deaminase or adenosine aminohydrolase	
**ARTN**	Artemin or enovin or neurobastin	X
**AXIN1**	Axin-1	X
**βNGF**	β-nerve growth factor	
**BDNF**	Brain-derived neurotrophic factor	X
**CASP-8**	Caspase 8	X
**CCL4**	Chemokine (C-C motif) ligand 4 or or macrophage inflammatory protein 1β (MIP-1β)	
**CCL11**	Chemokine (C-C motif) ligand 11 or eosinophil chemotactic protein or eotaxin-1	
**CCL19**	Chemokine (C-C motif) ligand 19 or EBI1 ligand chemokine (ELC) or macrophage inflammatory protein protein-3-β (MIP-3-β)	
**CCL20**	Chemokine (C-C motif) ligand 20 or liver activation regulated chemokine (LARC) or macrophage inflammatory protein-3 (MIP3A)	X
**CCL23**	Chemokine (C-C motif) ligand 23 or macrophage inflammatory protein 3 (MIP-3) or myeloid progenitor inhibitory factor 1 (MPIF-1)	X
**CCL25**	Chemokine (C-C motif) ligand 25	X
**CCL28**	Chemokine (C-C motif) ligand 28 or mucosae associated epithelial chemokine (MEC)	X
**CD5**	Cluster of differentiation 5	
**CD6**	Cluster of differentiation 6	X
**CD40**	Cluster of differentiation 40	
**CD244**	Cluster of differentiation 244	X
**CDCP1**	CUB domain-containing protein 1 or cluster of differentiation 318 (CD318) or transmembrane and associated with src kinases (Trask)	
**CX3CL1**	Chemokine (C-X3-C motif) ligand 1 or fractalkine	
**CXCL1**	Chemokine (C-X-C motif) ligand 1 or neutrophil-activating protein 3 (NAP-3)	
**CXCL5**	Chemokine (C-X-C motif) ligand 5 or epithelial-derived neutrophil-activating protein 78 (ENA-78)	
**CXCL6**	Chemokine (C-X-C motif) ligand 6	
**CXCL9**	Chemokine (C-X-C motif) ligand 9 or monokine induced by gamma-interferon (MIG)	
**CXCL10**	Chemokine (C-X-C motif) ligand 10 or interferon gamma-inducible protein 10 (IP-10) or small inducible cytokine B10	
**CXCL11**	Chemokine (C-X-C motif) ligand 11 or interferon-inducible T-cell α chemoattractant (I-TAC) or interferon-gamma-inducible protein 9 (IP-9)	
**CSF1**	Colony stimulating factor 1 or macrophage colony-stimulating factor (M-CSF)	
**CST5**	Cystatin D	
**DNER**	Delta and Notch-like epidermal growth factor-related receptor	
**EIF4EBP1**	Eukaryotic translation initiation factor 4E-binding protein 1	
**EN-RAGE**	Extracellular newly identified receptor for advanced glycation end-products binding protein or S100 calcium-binding protein A12 (S100A12) or calgranulin C	X
**FGF-5**	Fibroblast growth factor 5	
**FGF-19**	Fibroblast growth factor 19	

**FGF-21**	Fibroblast growth factor 21	X
**FGF-23**	Fibroblast growth factor 23	X
**Flt3L**	FMS-like tyrosine kinase 3 ligand	
**GDNF**	Glial cell-derived neurotrophic factor	X
**IFN γ**	Interferon γ	X
**HGF**	Hepatocyte growth factor or scatter factor (SF)	
**IL-1α**	Interleukin 1 α or hematopoietin 1	X
**IL-2**	Interleukin 2	X
**IL-2RB**	Interleukin 2 receptor subunit β	X
**IL-4**	Interleukin 4	X
**IL-5**	Interleukin 5	X
**IL-6**	Interleukin 6	X
**IL-7**	Interleukin 7	X
**IL-8**	Interleukin 8	
**IL-10**	Interleukin 10	X
**IL-10RA**	Interleukin 10 receptor α subunit or cluster of differentiation W210A (CDW210A)	X
**IL-10RB**	Interleukin 10 receptor β subunit or cluster of differentiation W210B (CDW210A)	
**IL-12B**	Interleukin 12 β subunit	
**IL-13**	Interleukin 13	X
**IL-15RA**	Interleukin 15 receptor α subunit	X
**IL-17A**	Interleukin 17 A	X
**IL-17 C**	Interleukin 17 C	X
**IL-18**	Interleukin 18 or interferon gamma inducing factor	
**IL-18R1**	Interleukin-18 receptor-1 or cluster of differentiation w218a (CDw218a)	
**IL-20**	Interleukin 20	X
**IL-20RA**	Interleukin 20 receptor α subunit	X
**IL-22 RA1**	Interleukin 22 receptor α1 subunit	X
**IL-24**	Interleukin 24	X
**IL-33**	Interleukin 33	X
**LIF**	Leukemia inhibiting factor	X
**LIFR**	Leukemia inhibiting factor receptor or cluster of differentiation 118 (CD118)	
**MCP-1**	Monocyte chemoattractant protein 1 or chemokine (C-C motif) ligand 2 (CCL2) or small inducible cytokine A2	
**MCP-2**	Monocyte chemoattractant protein 2 or chemokine (C-C motif) ligand 8 (CCL8)	
**MCP-3**	Monocyte chemoattractant protein 3 or chemokine (C-C motif) ligand 7 (CCL7)	X
**MCP-4**	Monocyte chemoattractant protein 4 or chemokine (C-C motif) ligand 13 (CCL13)	X
**MIP-1α**	Macrophage inflammatory protein 1-α or chemokine (C-C motif) ligand 3 (CCL3)	
**MMP1**	Matrix metalloproteinase 1 or interstitial collagenase or fibroblast collagenase	
**MMP10**	Matrix metalloproteinase 10 or stromelysin 2 or transin-2	
**NRTN**	Neurturin	X
**NT-3**	Neurotrophin 3	X
**OPG**	Osteoproteogerin	
**OSM**	Oncostatin M	X
**PDL1**	Programmed death-ligand 1 or cluster of differentiation 274 (CD274) or B7 homolog 1 (B7-H1)	
**SCF**	Stem cell factor or KIT-ligand (KL) or steel factor	
**SIRT2**	NAD-dependent deacetylase sirtuin-2	

**SLAMF1**	Signaling lymphocytic activation molecule 1 or cluster of differentiation 150 (CD150)	X
**ST1A1**	Sulfotransferase 1A1	X
**STAMBP**	STAM binding protein	X
**TGFα**	Transforming growth factor α	
**TGFβ1**	Transforming growth factor β 1	
**TNF**	Tumor necrosis factor or tumor necrosis factor α (TNFα) or cachectin	X
**TNFβ**	Tumor necrosis factor β or lymphotoxin	X
**TNFSF14**	Tumor necrosis factor superfamily member 14 or homologous to lymphotoxin, exhibits inducible expression and competes with HSV glycoprotein D for binding to herpesvirus entry mediator, a receptor expressed on T lymphocytes (LIGHT)	
**TNFRSF9**	Tumor necrosis factor receptor superfamily member 9 or cluster of differentiation 137 (CD137) or induced by lymphocyte activation (ILA)	
**TRAIL**	TNF-related apoptosis-inducing ligand	
**TRANCE**	TNF-related activation-induced cytokine or receptor activator of nuclear factor kappa-B ligand (RANKL) or tumor necrosis factor ligand superfamily member 11 (TNFSF11) or osteoprotegerin ligand (OPGL) or osteoclast differentiation factor (ODF)	X
**TSLP**	Thymic stromal lymphopoietin	X
**TWEAK**	TNF-related weak inducer of apoptosis or tumor necrosis factor ligand superfamily member 12 (TNFSF12)	
**uPA**	Urokinase type plasminogen activator	
**VEGFA**	Vascular endothelial growth factor A	

Figure S1. Scatter plots showing the NPX value of the proteins with a q-value < 0.05 in the comparison between RRMS and OND. The RRMS group is further splitted into MS OCB+ and MS OCB−. The bars indicate mean and standard deviations. (A) CD5, (B) IL-12B, (C) TNFB, (D) TNFSF14, (E) TNFRSF9, (F) MIP-1-alpha.

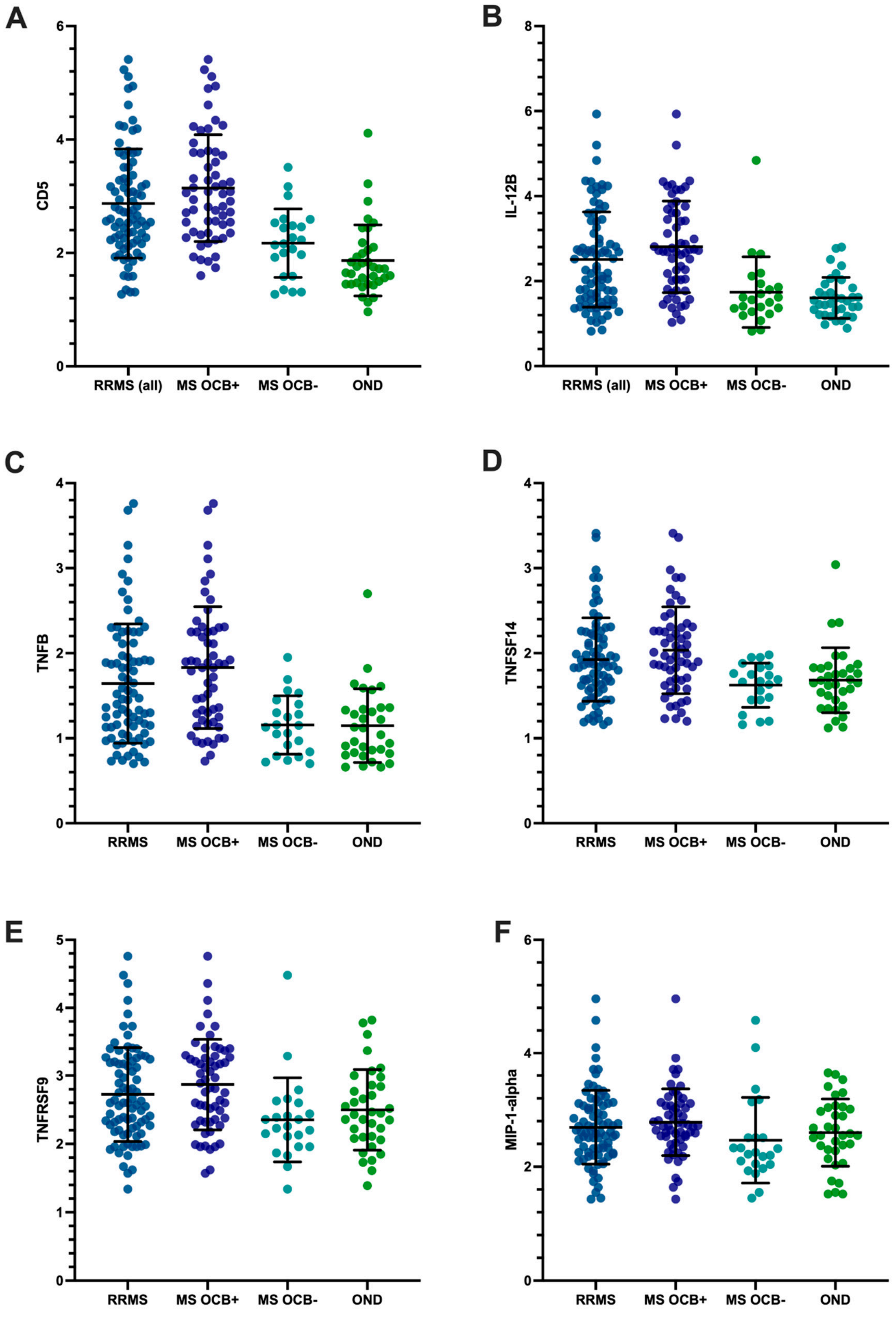

Figure legend. MS OCB+: multiple sclerosis with cerebrospinal fluid IgG oligoclonal bands. MS OCB—: multiple sclerosis without cerebrospinal fluid IgG oligoclonal bands. NPX: normalized protein expression. OND: other neurological diseases. PEA: proximity extension assay. RRMS: relapsing remitting multiple sclerosis.

Table S2. Coefficients relative to not z-scored NPX values in multivariate analysis of PEA-tested proteins in MS OCB+ vs. OND and MS OCB— vs. OND.

	**Coefficients**
**MS OCB+ vs. OND**	
**Intercept**	-5.68
**IL-12B**	1.24
**CD5**	0.92
**CX3CL1**	-0.20
**FGF-19**	-0.20
**CST5**	0.04
**MCP-1**	-0.13
**MS OCB— vs. OND**	
**Intercept**	2.2
**CX3CL1**	-1.04
**CD5**	0.42
**NfL**	0.00017
**CCL4**	0.15
**OPG**	-0.083

Table legend. MS OCB+: multiple sclerosis with cerebrospinal fluid IgG oligoclonal bands. MS OCB—: multiple sclerosis without cerebrospinal fluid IgG oligoclonal bands. NPX: normalized protein expression. OND: other neurological diseases. PEA: proximity extension assay.
